# Supplementary figures and images for: Biased synaptic activation of dentate granule cells by exercise reflects inputs from the lateral entorhinal cortex
Source: bioRxiv. 2026 Feb 23:2026.02.22.707119. Preprint. [Version 1] doi: 10.64898/2026.02.22.707119 (PMC13160126; doi:10.64898/2026.02.22.707119)

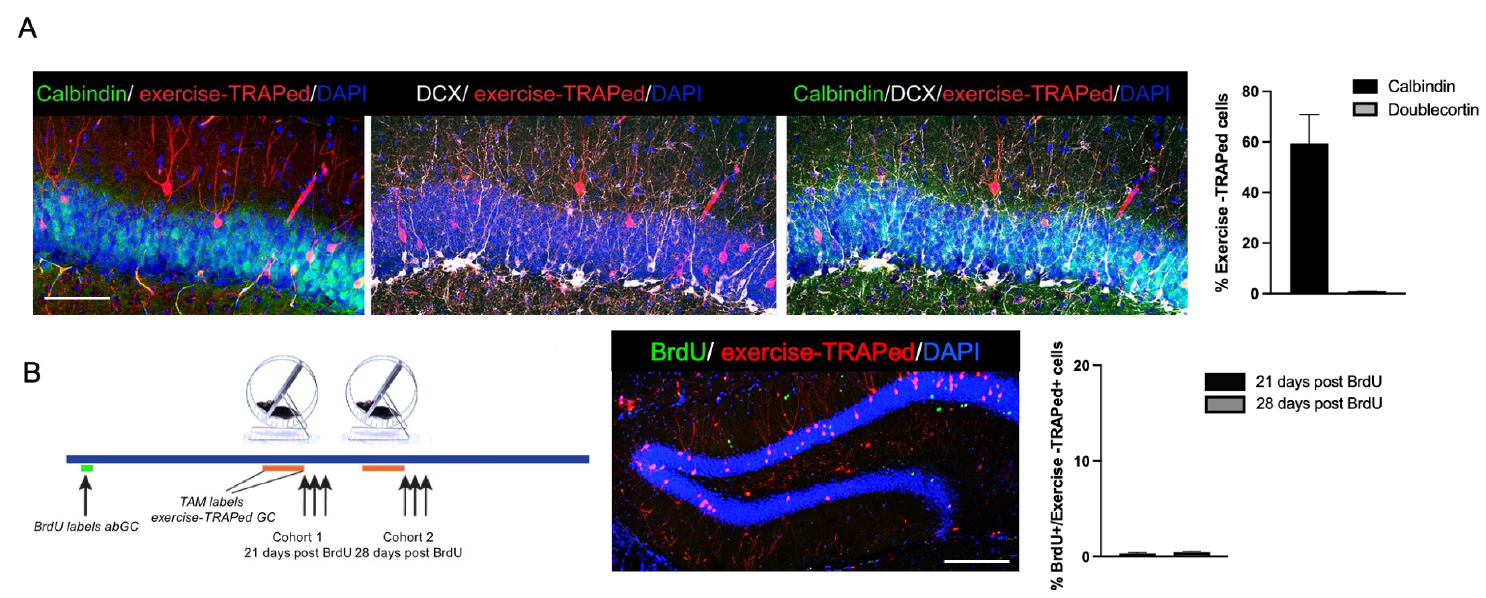

Supplement: Supplement 1 — Figure Supplemental 1. Exercise-TRAPed granule cells are mature granule cells. A. Representative images of exercise-TRAPed granule cells (red) co-stained for the mature granule cell marker calbindin (Cb) and the immature granule cell marker doublecortin (DCX) after 2hr of voluntary exercise, indicated that the exercise-TRAPed cells were mature (Cb+) granule cells. (Cb+/exercise-TRAPed+ overlap: 72.4 ± 5.2%; DCX+/ exercise-TRAPed overlap: 1.2% ± 0.01%, n=5, p < 0.0001). Scale bar = 100 μm. B. Experimental design for BrdU birth-labelling of exercise-TRAPed granule cells. Two-month-old male and female Fos-TRAP:TdTomato mice received two injections of BrdU per day (200mg/kg, i.p.). Mice were injected once with tamoxifen (150 mg/kg) 24 hr before exposure to 2 hr of voluntary exercise at 21 or 28 days post-BrdU. Mice were sacrificed 2 days after exposure to the running wheel. (Middle) Representative image of the dentate gyrus of Fos-TRAP:TdTomato mice 3 weeks after BrdU injection: BrdU (green), Exercise-TRAPed cells (red). See text for quantification. Scale bar = 200 μm. [file media-1.tif]
